# Are Alterations in DNA Methylation Related to CKD Development?

**DOI:** 10.3390/ijms23137108

**Published:** 2022-06-26

**Authors:** Jacek Rysz, Beata Franczyk, Magdalena Rysz-Górzyńska, Anna Gluba-Brzózka

**Affiliations:** 1 Department of Nephrology, Hypertension and Family Medicine, Medical University of Lodz, 113 Żeromskego Street, 90-549 Lodz, Poland; jacek.rysz@umed.lodz.pl (J.R.); bfranczyk-skora@wp.pl (B.F.); 2 Department of Otolaryngology, Laryngological Oncology, Audiology and Phoniatrics, Medical Univesity of Lodz, 113 Żeromskego Street, 90-549 Lodz, Poland; mrs-89@op.pl

**Keywords:** chronic kidney disease, epigenetics, DNA methylation

## Abstract

The modifications in genomic DNA methylation are involved in the regulation of normal and pathological cellular processes. The epigenetic regulation stimulates biological plasticity as an adaptive response to variations in environmental factors. The role of epigenetic changes is vital for the development of some diseases, including atherogenesis, cancers, and chronic kidney disease (CKD). The results of studies presented in this review have suggested that altered DNA methylation can modulate the expression of pro-inflammatory and pro-fibrotic genes, as well those essential for kidney development and function, thus stimulating renal disease progression. Abnormally increased homocysteine, hypoxia, and inflammation have been suggested to alter epigenetic regulation of gene expression in CKD. Studies of renal samples have demonstrated the relationship between variations in DNA methylation and fibrosis and variations in estimated glomerular filtration rate (eGFR) in human CKD. The unravelling of the genetic–epigenetic profile would enhance our understanding of processes underlying the development of CKD. The understanding of multifaceted relationship between DNA methylation, genes expression, and disease development and progression could improve the ability to identify individuals at risk of CKD and enable the choice of appropriate disease management.

## 1. Introduction

The prevalence of chronic kidney disease (CKD) is constantly increasing worldwide. The development of this disease is a prolonged process lasting several years. However, the occurrence of acute renal failure (as a result of toxic drug-induced tubular necrosis, ureter obstruction, contrast use or the thrombosis of the renal artery) could also evolve towards CKD. This disease is associated with inflammatory, metabolic, and cardiovascular diseases [1]. The advancement in research methods enabled the shift from single-gene studies toward genome-wide linkage studies [2]. Such studies involve the analysis of epigenetic risk factors. The knowledge of epigenetic mechanisms in the field of CKD origin is still in its infancy [2]. Nevertheless, several studies have presented the impact of epigenetic regulation, especially DNA methylation, on CKD. A better understanding of epigenetic changes in CKD would offer deep insight into disease pathophysiology, severity, and prognosis [2]. Epigenetic alterations mirror heritable changes which do not occur in the DNA sequence, which modify the phenotype, not the genotype [3]. In general, the term epigenetic modifications applies to the changes in DNA methylation, histone modifications, as well as regulatory RNA, including noncoding and long noncoding RNAs and microRNAs [4]. The epigenetic regulation stimulates biological plasticity as an adaptive response to variations in environmental factors [3]. DNA modifications, including altered DNA methylation, are vital transcription regulators altering gene expression and sometimes affecting disease progression or the occurrence of complications. The regions of actively transcribed genes are typically unmethylated or hypomethylated [5]. Tissues display a specific DNA methylation pattern which is established in the course of each organ development [6]. Since such a pattern is stable and has unique characteristics, it emerges as a good cell-specific biomarker. In contrast to RNA and proteins, which copy numbers or levels (respectively) depend on the magnitude of gene expression, DNA methylation status was found to directly mirror the cell number due to the fact that every cell contains one double DNA strand. Genome-wide variations in DNA methylation at CpG sites can be assessed using epigenome-wide association studies (EWAS) [7]. Some studies have observed altered DNA methylation patterns in patients with CKD [5,8,9].

## 2. Chronic Kidney Disease

Chronic kidney disease is considered an important cause of death worldwide and a chief factor contributing to cardiovascular morbidity and mortality [10,11,12]. According to the definition, CKD is related to the abnormalities of kidney structure or loss of renal function persisting for more than 3 months which is mirrored by decreased glomerular filtration rate (GFR) and/or other biomarkers, including proteinuria [13,14]. The prevalence of this disease rises from <5% in young adults to >40% among individuals ≥70 years [14]. The etiology of CKD involves inherited predisposition (genetic variants in the DNA sequence and/or epigenetic modifications) as well as exposure to environmental factors. Heritability has been estimated to account for 20–80% of interindividual differences in CKD [15]. A relatively high prevalence of CKD (approximately 10% of the general population) is associated with increased incidence of other diseases, such as hypertension, diabetes, glomerulonephritis and chronic pyelonephritis, lupus, and other autoimmune diseases, as well as repeated urinary infections in which progression is associated with the development of renal impairment or failure [1,16,17]. Diabetes mellitus and hypertension are considered to be the main causes of CKD (accounting for 60% of the cases) [18].

There are five distinct clinical stages of CKD determined on the basis of the patient’s serum creatinine, age, sex, and ethnicity (new recommendations suggest the development of endogenous filtration markers that would enable the elimination of racial and ethnic disparities) [15,19]. The first two stages of CKD are diagnosed in patients with eGFR below 90 mL/min/1.73 m^2^ (stage 1) or the range of 60–89 mL/min/1.73 m^2^ (stage 2) and signs of kidney dysfunction such as proteinuria. Subsequent stages are associated with more pronounced kidney impairment: eGFR = 30–59 mL/min/1.73 m^2^ (stage 3), eGFR = 15–29 mL/min/1.73 m^2^ (stage 4) and < 15 mL/min/1.73 m^2^ (stage 5). The presence of proteinuria and hypoxia is a vital trigger of epithelial-mesenchymal transition (EMT) initiation, probably via transforming growth factor-β1 (TGF-β1)/SMAD (suppressor of mothers against decapentaplegic) signaling [20]. Individuals having more advanced stages of CKD are at higher risk of cardiovascular disease. Moreover, progressive CKD may advance to end-stage renal disease, and such patients usually require chronic dialysis and/or renal transplantation [15].

The results of studies point out chronic inflammation as a factor implicated in the pathogenesis of various diseases, including CKD [21,22]. Inflammation also contributes to the progression of this disease [23]. The finding of diffuse interstitial infiltration of neutrophils, T lymphocytes, B lymphocytes, and monocytes within the kidney seems to confirm the importance of the aforementioned process. It was observed that the infiltrated leukocytes release cytokines and growth factors, thus creating an inflammatory milieu. At the same time, they also produce anti-inflammatory and pro-regenerative cytokines in order to cease inflammation and facilitate renal tissue repair [24]. Transient recruitment of immune cells is beneficial, however, prolonged accumulation of leukocytes within the renal interstitial compartment triggers chronic inflammation and results in renal fibrosis [25]. Tubulointerstitial renal fibrosis inevitably leads to renal function deterioration, independently of underlying renal disease [20]. Renal fibrosis is a common histopathological feature of CKD, while the presence of tubulointerstitial fibrosis is considered a strong marker of CKD progression [26].

The biological mechanisms related to CKD and its progression remain elusive [7]. Currently, the treatment of chronic kidney disease involves the management of common abnormalities, including proteinuria, hyperglycemia, hypertension, etc., however, in some patients these measures are not sufficient and thus they progress to end-stage renal disease (ESRD) [23]. Genome-wide association studies (GWAS) and epigenome-wide association studies (EWAS) have identified frequent variants within more than >400 genetic loci that are associated with renal function and the development of CKD [27,28,29,30]. Moreover, large EWAS including participants of European and African American origin revealed 19 CpGs in whole blood that affected eGFR or CKD [7]. It is estimated that genetic variants are responsible for ~8.9% of eGFR variance in CKD [29]. The rate of premature deaths among patients with CKD is high as a result of complications (e.g., cardiovascular disease, anemia). The incidence of complications in this group of patients correlates with changed DNA methylation patterns and subsequent dysregulated expression of genes subjected to such regulation [1]. Therefore, the analysis of epigenetic modifications is needed to uncover the mechanisms underlying the worsening of eGFR and the onset of CKD.

## 3. DNA Methylation

**Figure 1:**
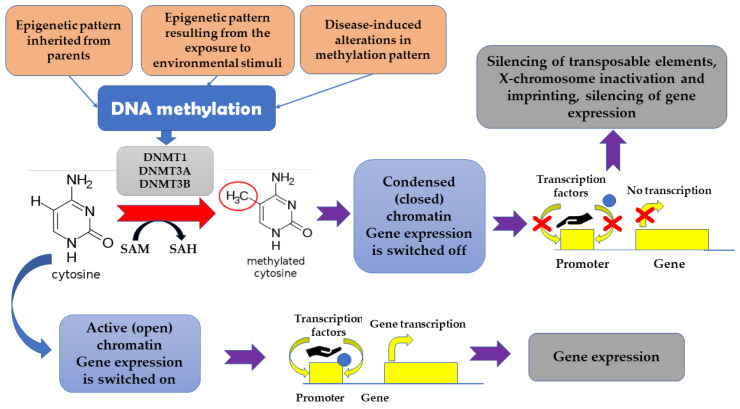
The impact of methylation on gene expression.

The term “epigenetics” denotes heritable dynamic modifications in the expression of genes which are associated with factors beyond changes in DNA sequence. Such changes can be inherited from parents, but also can be a result of exposure to some environmental stimuli, including drugs, diseases, or diet [15]. It appears that epigenetic changes mirror dynamic associations between an individual’s genetic background and important environmental stimuli [9]. DNA methylation was found to be associated with cell type-specificity [31]. Generally, genome-wide pattern of DNA methylation appears to be conserved across many species. The highest epigenetic conservation is observed in relation to gene regulatory elements and active chromatin modifications. It has been suggested that the epigenetic maintenance and turnover of transcription factor binding sites may serve as a trigger associated with tissue-specific DNA methylation [31]. It is estimated that 60–90% of CpG (cytosine-phosphate-guanine) sites in the mammalian genome are methylated, which results in the presence of 5-methyl-cytosine in these sites [20]. Unmethylated areas are observed primarily in CpG islands which, in turn, are frequently located within the promoters of housekeeping genes and actively transcribed genes [20]. The methylation of DNA reversibly affects gene expression without modifying DNA sequence [32,33]. DNA methylation-related transcriptional downregulation was suggested to be associated with hampering the interaction between transcription factors and their targets as well as with the recruitment of specific transcriptional repressors to the methylated DNA [23]. Apart from being involved in the silencing of transposable elements, X-chromosome inactivation and imprinting, DNA methylation is a powerful tool responsible for transcriptional silencing. DNA methyltransferase 1 (DNMT1), as well as DNMT3A and DNMT3B, are involved in re-establishing of methylation patterns and de novo methylation since they catalyze the addition of a methyl group to the cytosine [34,35]. In turn, the reduction of 5mC to 5-hydroxy-methyl-cytosine (5hmC) is associated with the activity of Ten-Eleven Translocation (TET) proteins [36]. The silencing of genes appears to be associated with the change of chromatin conformation, which make such genes inaccessible for factors required for transcription. Additionally, proteins, such as methyl-CpG binding domain (MBD) proteins, attach to the methylated DNA, thus hampering the transcription. In some cases, DNA methylation can result in the activation of some genes. It happens when the DNA methylation directly hinders those cofactors or miRNA which normally suppresses the transcription [15] (Figure 1).

## 4. DNA Methylation in CKD

Epigenetics has been postulated to mediate the effects of environmental factors on renal development [20]. Several epigenetic modifiers have been suggested to participate in renal development (e.g., transcription factor Pax2, histone deacetylases HDAC1 and HDAC2), however, their exact role remains elusive [37,38,39]. However, this field has been poorly characterized.

The modifications in genomic DNA methylation have been found to be involved in the regulation of normal and pathological cellular processes [40]. Epigenetic changes are vital for the development of some diseases, including atherogenesis, cancers, and CKD [41,42]. Disturbed metabolic state, such as uremia in CKD could result in the modification of epigenetics-mediated gene expression. In such a manner, uremic “memory” is established [43,44]. The results of studies have demonstrated that epigenetic “memory” of the earlier trigger can be maintained for a long time and modulate the forthcoming gene expression profile [23]. A complex system comprising epigenetic regulators and transcription factors regulates altered gene expression in CKD [20]. Some differentially methylated regions were demonstrated to be enriched for genes involved primarily in development and no longer expressed in the kidneys of an adult individual, which may suggest the establishment of epigenetic changes in the period of organ development. Such a thesis may provide a possible connection between fetal programming and CKD development. According to studies, DNA methylation affects CKD development, but also CKD may induce alterations in methylation patterns [45]. Ko et al. [46] suggested the existence of the causal relationship between cytosine methylation differences and both transcript levels and phenotype development. They found that various forms of CKD had consistent differences in cytosine methylation which translated into baseline levels of gene expression [46]. Similarities in changes in cytosine methylation and gene expression in tubule samples collected from hypertensive and diabetic individuals indicate a common mechanism of CKD progression. Moreover, they observed that the majority of methylation differences were present outside promoter areas, primarily at candidate enhancers. Furthermore, they demonstrated the presence of consensus-binding motifs for crucial renal transcription factors such as SIX2, HNF, and TCFAP within differentially methylated regions. These genes are considered essential for CKD development [47,48].

### 4.1. Homocysteine

The results of many studies have demonstrated that abnormally increased homocysteine (Hcy) accelerates the progression of CKD via the modulation of oxidative stress, endoplasmic reticulum stress, and inflammation [49,50]. Ding et al. [50] demonstrated that higher homocysteine levels downregulated the expression of miR-30a-5p in mice and Hcy-treated podocytes. 3′-untranslated region (3′-UTR) of the forkhead box A1 (FOXA1) is the target of miR-30a-5p, thus overexpression of this miRNA is associated with the inhibition of FOXA1 expression [50]. Moreover, they suggested that the downregulation of miR-30a-5p mediated in Hcy-induced glomerular podocyte injury in a DNA methylation-dependent manner. The overexpression of miR-30a-5p was found to inhibit the development and progression of glomerular podocyte injury induced by Hcy via targeting FOXA1. In turn, DNA hypermethylation promoted Hcy-induced podocyte injury via a mechanism involving the downregulation of miR-30a-5p expression [50]. Ingrosso et al. [51] demonstrated that hyperhomocysteinemia altered epigenetic regulation of gene expression since they observed higher DNA hypomethylation in peripheral mononuclear cells compared to controls. Their finding implies that the toxic action of homocysteine could involve macromolecule hypomethylation. Moreover, they reported that folate therapy not only decreased hyperhomocysteinemia in their study group but also restored DNA methylation to normal levels as well as improved gene expression patterns [51]. Hyperhomocysteinemia is common in patients with renal failure and it seems that it can also affect the DNA methylation pattern [52]. Additionally, Yang et al. [53] observed that hyperhomocysteinemia could trigger glomerular damage through mechanisms involved in oxidative stress and the impairment of DNA methylation. It has been suggested that methionine–homocysteine can regulate both the S-Adenosylmethionine (SAM)/S-Adenosyl-L-homocysteine (SAH) ratio and the rate of SAM-dependent methylation under pathological conditions [1]. A clinical trial of men with hyperhomocysteinemia and uremia receiving standard hemodialysis treatment demonstrated the positive impact of folate administration on the epigenetic control of some imprinted genes [51]. In their study, the expression of insulin-like growth factor 2 (IGF-2) (silenced in patients from the study group) and H19 (displaying biallelic expression) was restored in a normal monoallelic manner with an overall rise in DNA methylation levels. Therefore, the authors concluded that deficient folate status could affect the epigenetic regulation of gene expression, while the supplementation of foliate could reverse this process.

### 4.2. Hypoxia

The progression of acute kidney injury to CKD can be induced by hypoxia via epigenetic mechanisms promoting fibrosis and inflammation [54]. Under hypoxic conditions, hypoxia-inducible factor 1 (HIF-1) modulates the expression of downstream targets, partly via epigenetic mechanisms [55]. HIF-1 is an oxygen-sensitive transcriptional activator which regulates cellular adaptation to low oxygen and nutrient-deprived environment [56]. In contrast to normoxic conditions in which HIF-1 undergoes degradation, in hypoxia, HIF-1 accumulates and stimulates the expression of the histone demethylases (JMJD1A and JMJD2B), thus promoting reduced histone methylation and changed expression of downstream genes [57,58]. It controls the expression of oxygen-regulated genes, including (EPO), via CpG methylation of the DNA-binding site for HIF-1 [1,59]. This factor may trigger alterations in chromatin conformation, thereby affecting genes transcription [60]. According to Sato et al. [61], CKD-related anemia is associated with the impairment of the mechanism connecting the presence of hypoxia and the synthesis of EPO. The methylation of CpG island surrounding gene promoter and a 5′-untranslated region (5′-UTR) was found to silence the expression of erythropoietin, while the use of DNMT1 inhibitor (5-aza-2′-deoxycytidine) could restore EPO production in primary mouse myofibroblast cultures [62,63]. EPO is an erythroid growth factor necessary for erythropoiesis, the lack of which is associated with anemia. Due to the fact that kidneys are the chief organ producing erythropoietin, in CKD patients erythropoiesis is frequently disabled [64,65]. In anemia, EPO is also synthesized by the liver, but it cannot compensate for impaired renal production [61]. Apart from HIF-1, the gene promoter for *HIF2α* was demonstrated to be highly methylated, resulting in the silencing of its expression and lack of the protein, even in hypoxia [61].

### 4.3. Inflammation

DNA methylation was found to be involved in the development of chronic kidney inflammation which is a common process in CKD irrespective of the underlying cause of the disease (hypertension, hyperglycemia, chronic infection, and autoimmune disorder) [23]. The presence of these stimuli could markedly modify the DNA methylation pattern of circulating immune cells, which results in the upregulation of pro-inflammatory genes and the development of inflammatory state. Stenvinkel et al. [40] demonstrated the relationship between global DNA methylation and an inflammation in dialysis patients. In their study based on the LUMA technique, a lower HpaII/MspI ratio was indicative of higher global DNA methylation [40,66]. Inflammation in dialysis patients was defined there as C-reactive protein (CRP) ≥10 mg/L. Markedly lower HpaII/MspI ratios in PBC were demonstrated in the presence of inflammation compared to individuals without signs of inflammation. Moreover, the DNA inflammation correlated also with other inflammatory biomarkers, such as procalcitonin (a marker of bacterial infection) and ferritin [40]. Thus, the authors suggested that altered methylation may be stimulated by a subclinical bacterial infection in dialysis patients. The analysis of the impact of worsening renal function and chronic inflammation on the DNA methylation and outcomes of patients suggested that inflammation may drive abnormal DNA hypermethylation and that DNA hypermethylation affected both all-cause and cardiovascular mortality in CKD stage 5 patients [40]. The authors suggested that epigenetic modifications may contribute to accelerated atherosclerosis in this group of patients. It appears that inflammatory cytokine IL-6 may be involved in the regulation of DNA methyltransferase gene, thus triggering epigenetic changes in cells [67]. The results of in vitro study implied that IL-6 could sustain promotor methylation via the increase in the expression of DNMT-1 [68]. Smyth et al. [9] observed higher DNA methylation, especially for pro- and anti-inflammatory markers in CKD patients compared with controls without renal disease. The results of the study performed by Wing et al. [5] supported the role of inflammation in the progression of CKD since they demonstrated the correlation between inflammatory biomarkers and renal function and proteinuria. The Chronic Renal Insufficiency (CRIC) Study revealed the correlation between altered methylation of genes which protein products were involved in inflammation and oxidative stress (e.g., *CLU, NFKBIB, NFKBIL2, NOS3, TGFBI*, and *TGFB3*) and the rate of renal function loss [5]. NKFBIL2 belongs to the IκB family and functions as NF-κB inhibitor in the nucleus, while the second one, NFKBIB, acts as a transcriptional activator as well as a chaperone protecting IκB from losing its activity [69,70].

### 4.4. DNA Methylation and Renal Fibrosis

Studies of renal samples have demonstrated the relationship between variations in DNA methylation and fibrosis in human CKD [71,72,73]. Ko et al. [46] indicated enhanced expression of profibrotic genes: *TGFBR3, SMAD3, SMAD6* in tubule from CKD patients. SMAD3 protein is one of the most crucial mediators of the pro-fibrotic effects associated with both TGFβ and angiotensin II pathways [46,48]. In another study, 5 CpGs (within or in the vicinity of PTPN6/PHB2, ANKRD11, TNRC18, PQLC2, and PRPF8) in kidney cortex samples correlated with the degree of fibrosis [7]. Hypermethylation in the aforementioned sites translated into better kidney function and lower kidney fibrosis. Recent evidence for the involvement of the epithelial-mesenchymal transition (EMT) process was found in renal fibrosis [5]. Fibrosis is associated with the switch of a differentiated epithelial cell into matrix-producing fibroblasts and myofibroblasts. Many studies have demonstrated the role of epigenetic changes in the regulation of EMT [74,75,76]. The results of studies have suggested that the upregulation of DNMT1, DNA methylation, and transcriptional silencing are involved in fibroblast activation and EMT in kidney fibrosis [76]. Moreover, the study of renal disease models demonstrated that the hypermethylation of the *RASAL1* promoter and consequently decreased transcription of this gene was associated with acute kidney injury (AKI) and chronic progressive fibrosis [76]. Additionally, the study of an animal model of renal fibrosis demonstrated hypermethylation of the *RASAL1* gene. *RASAL1* encodes Ras GTPase-Activating-Like Protein 1, which is a suppressor of RAS function and enables the control of cellular proliferation and differentiation [76]. As a result of *RASAL1* hypermethylation, reduced expression of its protein product is observed. Such hypermethylation correlates with the presence of activated fibroblasts following the regression of acute renal injury. The finding that fibrotic mice kidneys displayed the expression of DNMT1, but not DNMT3a or DNMT3b in comparison to control kidneys, may imply the involvement of DNMT1 in the methylation of *RASAL1* as well as in the progression of kidney fibrosis [2]. The role of *RASAL1* hypermethylation in the pathogenesis of kidney fibrosis has been proved in other models of nephropathy, ureteral obstruction, and nephrotoxic serum nephritis [76,77,78]. Chronic Renal Insufficiency (CRIC) Study revealed the correlation between altered methylation of genes which protein product were involved in EMT and fibrosis (e.g., *NPHP4, IQSEC1*, and *TCF3*) and the rate of renal function loss [5]. Nephronophthisis 4 (NPHP4), transcription factor 3 (TCF3), and IQ motif and Sec7 domain 1 (IQSEC1) were found to be involved in pathways that promote the epithelial to mesenchymal transition and renal fibrosis. Therefore, their relevance for the progression of renal disease was suggested [5]. *NPHP4* gene encodes a protein that functions in a complex which plays an important role in cell–cell and cell–matrix adhesion signaling [79]. Impairment of NPHP4 was found to be associated with renal fibrosis and cystogenesis in nephronophthisis [80]. In turn, IQSEQ1 activates ADP-ribosylation factor 6 (ARF6), thus contributing to the regulation of actin cytoskeleton and cell adhesion. Hiroi et al. [81] demonstrated that the overexpression of IQSEQ1 in rat kidney cells was associated with the disappearance of F-actin. TCF3 is a transcription factor E2-alpha which activates transcription via the binding to regulatory E-box sequences on target genes. Along with TCF15, TCF3 is necessary for the mesenchymal to epithelial transition. Its suppression was indicated by modifications of cell morphology and cytoskeletal arrangement in the process of TGF-β-controlled EMT [5,82]. The hypermethylation of *IQSEC1, NPHP4,* and *TCF3* was significantly higher in stable renal function compared to patients with rapid loss of eGFR [5]. The majority of the methylation differences were noticed in candidate enhancers rather than in the promoter regions of genes [9]. The results of another study have demonstrated that TGFB1 signaling could be involved in the loss of epithelial cell adhesions as well as the damage of tubular basement membrane, thus contributing to renal fibrosis [74]. Similarly, there are reports indicating the role of TGFB3 in the promotion of renal cells fibrosis [83].

### 4.5. DNA Methylation Impact on eGFR

eGFR-related DNA methylation displayed enrichment for localization in binding sites of the transcription factors: CCAAT Enhancer Binding Protein Beta (CEBPB), early B cell factor 1 (EBF1), and E1A Binding Protein P300 (EP300) [7]. The study of the animal model demonstrated that transcription factor EBF1 was crucial for the formation of the glomerular tuft [84]. In turn, the absence of EBF1 within the glomerular mesangium resulted in the impaired development of glomeruli. In mesangial cells, EBF1 regulates glomerular capillary branching via the stimulation of nuclear factor of activated T-cells (NFAT) and subsequently increased the expression of cyclooxygenase 2 (COX-2) [84]. Based on the obtained results, it has been suggested that EBF1 regulates the expression of genes, the products of which are essential for both kidney development and function. Therefore, it is plausible that alterations in EBF1 gene methylation may be related to kidney damage. In turn, Schlosser et al. [27] identified changes in methylation patterns in 69 CpGC related to eGFR, and 65 of them were also observed in CKD patients. Moreover, the methylation of CpGs cg18194850 in *SUCLG2* gene and cg07242931 in *MAN1C1* gene correlated not only with eGFR but also with time to kidney failure or acute kidney injury [27]. The *SUCLG2* (Succinate-CoA Ligase GDP-Forming Subunit Beta) gene, mostly expressed in kidneys, liver, heart, spleen, and skeletal muscle, encodes β-subunit of succinyl-CoA synthetase; this synthetase catalyzes reversible reaction leads to the formation of succinyl-CoA and succinate. Genetic variations in this gene were found to be associated with urinary levels of succinyl-carnitine, while its higher blood levels correlated with lower eGFR [85]. In turn, two CpGs at the solute carrier family 1 member 5 (*SLC1A5*) were demonstrated to affect gene expression and blood pressure. *SLC1A5* encodes a sodium-dependent neutral amino acid transporter. DNA methylation within *SLC1A5* was suggested to be the possible link between urinary albumin-to-creatinine ratio (UACR) and blood pressure [27]. One epigenome-wide association study (EWAS) aiming to identify additional CpGs related to gene regulatory mechanisms involved in CKD found that expression alterations induced by DNA methylation did not always apply to the closest gene [27]. Sometimes, the CpG site correlated with multiple genes in cis. Schlosser et al. [27] also observed the impact of DNA methylation at cg04864179 on *IRF5* transcript levels, which might subsequently affect eGFR. Colocalization of *IRF5* gene expression with eGFR has been reported in blood as well as tubular and glomerular compartments of kidney tissue [86]. IRF5 is a transcription factor, a member of the interferon regulatory factor (IRF) family. This factor is vital for innate immunity since it promotes the expression of type I interferon (IFN) IFNA and INFB as well as inflammatory cytokines downstream of toll-like receptors TLR7, TLR8, and TLR9 [87]. Moreover, IRF5 can be involved in the regulation of apoptosis, growth, and differentiation. Several other studies have also reported that the methylation of IRF5 can alter kidney function while acting through immune pathways (e.g., systemic lupus erythematosus (SLE)) [88,89,90]. Despite the fact that DNA methylation is cell-specific, Schlosser et al. [27] found similar eGRF-related CpGs patterns of DNA from blood and renal tissue, which implies that some findings from blood may mirror those of the target organ. The enrichment analyses of CpGs associated with renal function revealed the vital role of transcriptional regulation since the enrichment of primed and active enhancers H3K4me1/3 and H3K36me3, as well as enrichment of DNA-Directed RNA Polymerase II Largest Subunit (POLRA2), have been observed. POLRA2 is the fundamental constituent of the RNA polymerase II transcription machinery that is responsible for the synthesis of mRNA in eukaryotes.

Wing et al. [5] revealed 15 CpG sites with a higher degree of hypermethylation in the stable kidney function group compared with rapid progressors. CpGs within clusterin (*CLU*), nitric oxide synthase 3 (*NOS3*), transforming growth factor beta 3 (*TGFB3*), cysteinyl-tRNA synthetase 2, mitochondrial (*CARS2*), *DNMT3A*, DNA methyltransferase 3 alpha (*TGFBI*), tonsoku like, DNA repair protein (*NFKBIL2*), and NFKB inhibitor beta (*NFKBIB*) genes were reported to be hypermethylated in the stable kidney function group compared to individuals with rapidly progressing renal disease [5]. The identified CpG sites were located within genes associated with the development and function of renal tubules, the regulation of actin cytoskeleton remodeling and cell adhesion, as well as the suppression and regulation of peroxisomes at a transcriptional level. Hypermethylation of CpGs within the *CLU* gene was markedly more pronounced in the stable kidney function group. It appears that methylation patterns in individuals with rapidly progressing kidney disease may be associated with higher expression of CLU since greater CLU expression correlates with loss of kidney [91].

### 4.6. Other

Genome-wide quantitative analysis of 485,577 DNA methylation sites in blood collected from 407 revealed altered methylation of CpG islands of the following genes: cut-like homeobox 1 (*CUX1)*, engulfment and cell motility 1 (*ELMO1*), FK506-binding protein 5 (*FKBP5)*, inhibin-βA-AS1 (*INHBA-AS1)*, protein tyrosine phosphatase receptor type N polypeptide 2 (*PTPRN2*), and *PRKAG2* genes [9,15]. CUX1 (Cut Like Homeobox 1) belongs to the homeodomain family of DNA binding proteins [92]. It was suggested to be involved in the regulation of gene expression, morphogenesis, differentiation, as well as cell cycle progression. CUX1 plays a vital role in kidney development. Its abnormal expression was associated with kidney disease [92,93]. *ELMO1* gene encodes the engulfment and cell motility protein 1. The results of studies indicate its involvement in the development of diabetic kidney disease [94,95]. ELMO1 was found to interact with dedicator of cytokinesis proteins (Dock180) and small GTPase Rac1, affecting cell migration and stimulating phagocytosis [94]. Reduced methylation of *ELMO1* was reported in patients with CKD compared to controls. Lower methylation translated into a higher level of *ELMO1* gene expression [9]. Higher expression of this protein was suggested to result in aberrant regulation of the extracellular matrix and consequently lead to kidney damage [96]. Another study demonstrated the association between increased expression of ELMO1 and the enhanced production of extracellular protein, diminished cell adhesion, as well as the accelerated progression of T2DM glomerulosclerosis [97]. *FKBP5* encodes a cytosolic chaperone FK506 binding protein 51 (FKBP5), which acts as a negative regulator of GR signaling [98]. Epigenetic control of FKBP5 is involved in the regulation of stress hormones [9]. The results of studies suggested that diminished methylation at this *FKBP5* locus was associated with enhanced stress-dependent gene transcription [99]. Another study observed aberrant DNA methylation within *FKBP5* in individuals with CKD, which implies the possible role of FKBP5 in the development and progression of CKD [9]. Wilson et al. [100] suggested that *FKBP5* hypermethylation may be associated with decreased chromatin accessibility in GR response elements and diminished activity of the glucocorticoid receptor-negative feedback loop. *INHBA-AS1* encodes a long non-coding antisense RNA [9]. According to some studies, this RNA could be intricated in kidney damage and transplant rejection [101,102]. In turn, PRKAG2 acts as a vital regulator of metabolic functions [9]. Polymorphisms within this gene were associated with enlarged kidneys, serum creatinine levels, and CKD [103,104]. Moreover, it has been found that gene expression of *PRKAG2* gene differed significantly between individuals with CKD and controls [105]. Finally, *PTPRN2* encodes islet antigen (IA)-2β which, together with IA-2, functions as an autoantigen associated with type 1 diabetes mellitus (T1DM) [106,107,108]. Variations within its gene were associated with CKD [109]. Another study revealed the relationship between dmCpGs within *PTPRN2* and T1DM-ESKD [108]. Some studies indicated that enhanced renal expression of *PTPN6* (encoding Protein Tyrosine Phosphatase Non-Receptor Type 6, known also as Src homology-2 domain-containing phosphatase-1 (SHP-1)) was associated with renal disease and vascular complications in diabetes [110,111].

The analysis of methylation patterns in 6 various genes revealed markedly decreased DNA methylation of *COLIVA1* (collagen of the basement membrane, alpha-1 chain) and *COLIVA2* in CKD compared control group. These genes encode vital basement membrane proteins. The results of studies have indicated that their enhanced expression was associated with the thickening of the basement membrane, a phenomenon observed in the early stages of progressive kidney fibrosis [112]. *COLIVA1 and COLIVA2* share a promoter. In CKD, this region contained a considerably lower absolute methylation level (by about 50%) compared to controls. Most differentially methylated regions occurred within gene body-related regions and primarily in intronic regions [46]. Differences in methylation translated into a higher amount of *COLIVA1* transcript and its protein.

## 5. Potential Therapies

The observation that DNA methylation can be reversed by TET enzymes provides new potential therapies for CKD and other diseases [77,113]. Additionally, a deep comprehension of DNMTs functions in different states might help to design effective strategies to restore immune homeostasis and hamper the progression of some diseases. Many clinical trials assess the efficiency of demethylating drugs eliminating abnormal methylation of promoter regions, however, the majority of them focus on cancers [20]. However, the results of some studies indicate that pharmacological intervention targeting aberrant epigenetic changes could slow down the progression of renal fibrogenesis [20]. The targeting of DNA methylation (e.g., hydralazine) in animal studies was found to decrease renal inflammation and fibrosis in animals with progressive CKD [78,114]. Several studies demonstrated that the administration of 5-azacytidine (demethylation agent) improves tubulointerstitial fibrosis in an experimental model of renal fibrosis [76,115]. However, due to the fact that 5-azacytidine and its derivate 5-aza-2-deoxycytidine could also affect the methylation of regularly methylated genes, their use may be associated with severe side effects. In turn, the administration of bone morphogenic protein 7 (BMP7) in experimental kidney fibrosis resulted in Tet3-mediated reversal of aberrant hypermethylation at the *Rasal1* promoter, as well as the hampering of kidney fibrosis [77]. TET-mediated hydroxymethylation is involved in the demethylation of active DNA to restore gene expression [113]. Moreover, the use of low doses of hydralazine in a mouse model of ischemia-reperfusion injury was associated with hampered progression into renal fibrosis as well as conserved excretory renal function [78]. Unfortunately, currently, the knowledge of alterations of the epigenome is insufficient, and the majority of pharmacological agents targeting such changes are cytotoxic.

Since the question of whether the reversal of alterations in DNA methylation patterns could improve renal dysfunction and disease progression is still open, further large studies are required to solve this puzzle. The examples of genes which altered methylation and suggested to be involved in CKD development are presented in Table 1.

## 6. Conclusions

The results of the aforementioned studies have provided evidence for the role of the alterations in DNA methylation in the modulation of the expression of inflammatory, pro-fibrotic, and other genes, thus stimulating renal disease progression [20]. Modifications of DNA methylation pattern can precede alterations in kidney function or the development of CKD, but also be their consequence [7]. The knowledge of aberrant DNA modifications would enable the identification of patients who are at a higher risk of developing CKD. Moreover, the use of epigenetic biomarkers associated with CKD could enhance the understanding of the biological mechanisms underlying this disease. The question of whether the reversal of alterations in DNA methylation pattern could improve renal dysfunction and disease progression is still open, thus further large studies are required to solve this puzzle.

## Figures and Tables

**Table 1:** Examples of genes which altered methylation and were suggested to be involved in CKD development.

Differently Methylated Region/Gene(s)	Effect	Ref
miR-30a-5p promoter	Increased methylation level inhibits its expression. DNMT1-mediated DNA hypermethylation promotes Hcy-induced podocyte injury by downregulation of miR-30a-5p expression.	[1]
*NPHP4*	Protein involved in renal tubular development and function Gene hypermethylation—silencing of gene expression Disrupted NPHP4 function may contribute to renal fibrosis and cystogenesis in nephronophthisis. Hypermethylation was significantly higher in stable renal function compared to patients with rapid loss of eGFR.	[2]
*IQSEC1*	Interacts and activates ARF6 for regulation of cell adhesion and actin cytoskeleton. Gene hypermethylation—silencing of gene expression Hypermethylation was significantly higher in stable renal function compared to patients with rapid loss of eGFR.	[2]
*TCF3*	Gene hypermethylation—silencing of gene expression Suppression leads to TGF-β-controlled EMT changes: expression of α-smooth muscle actin and E-cadherin, and alterations in cell morphology and cytoskeletal arrangement. Hypermethylation was significantly higher in stable renal function compared to patients with rapid loss of eGFR.	[2,3]
*CLU*	higher degree of hypermethylation in the stable kidney function group compared with the rapid progressors (*p* = 0.010).	[2]
*NFKBIL*	Inhibitor of NF-κB—stops the activation of target genes in the nucleus. Hypermethylated in the stable kidney function.	[2]
*NFKBIB*	Transcriptional activator of NF-κB. Hypermethylated to a larger extent in the stable kidney function group.	[2]
*TGF-β3*	Promotes fibrosis in renal cells, mainly mediated through TGFB1 pathways. CpGs for the gene were hypermethylated to a larger extent in the stable kidney function group compared with the rapid progressors.	[2]
*TGFB1*	TGFB1 signaling: loss of epithelial cell adhesions and disruption of the tubular basement membrane, leading to renal fibrosis. CpGs for the gene were hypermethylated to a larger extent in the stable kidney function group compared with the rapid progressors.	[2]
*RASAL1* promoter	RASAL1 protein is a suppressor of RAS function—the control of cellular proliferation and differentiation. Hypermethylation decreased transcription of this gene and was associated with acute kidney injury (AKI) and chronic progressive fibrosis.	[14]
*EBF1*	Regulates the expression of genes which products are essential for both kidney development and function. Altered methylation may be related to kidney damage.	[84]
*IRF5*	Factor crucial for innate immunity (it stimulates the expression of IFNA and INFB and inflammatory cytokines downstream of toll-like receptors TLR7, TLR8, and TLR9) and involved in the regulation of apoptosis, growth, and differentiation. Its methylation may alter kidney function acting through immune pathways (e.g., systemic lupus erythematosus (SLE)).	[26,27,28,29]
*CUX1*	Involved in the regulation of gene expression, morphogenesis, differentiation, as well as cell cycle progression in kidney development. Its abnormal expression was associated with kidney disease.	[9,15,34,35]
*ELMO1*	Involved in the development of diabetic kidney disease. Affects cell migration and phagocytosis. Lower methylation—higher level of *ELMO1* gene expression. Reduced methylation in patients with CKD compared to controls. Higher expression—aberrant regulation of the extracellular matrix and kidney damage	[5,36,37,38]
*FKBP5*	Negative regulator of GR signaling. Epigenetic control involved in the regulation of stress hormones. Diminished methylation—enhanced stress-dependent gene transcription.	[5,40,41]
*INHBA-AS1*	Long non-coding antisense RNA. Possibly involved in kidney damage and transplant rejection.	[5,43,44]
*PRKAG2*	Vital regulator of metabolic functions. Gene expression differed significantly between individuals with CKD and controls.	[5,47]
*COLIVA1*, *COLIVA2*	Vital basement membrane proteins. Markedly decreased DNA methylation (higher amount of transcript and protein) in CKD compared control group. Enhanced expression—the thickening of the basement membrane in the early stages of progressive kidney fibrosis.	[54]

+/− heterozygous knockout; 5′-UTR—5′-untranslated region; ARF6—ADP-ribosylation factor 6; Cbs—cystathionine beta-synthase; CLU—clusterin; COLIVA1—collagen of the basement membrane, alpha-1 chain; CUX1—cut-like homeobox 1; ELMO1—engulfment and cell motility 1; EMT—epithelial to mesenchymal transition; FKBP5—FK506-binding protein 5; GR—glucocorticoid; Hcy—homocysteine; type I interferon (IFN); INHBA-AS1—inhibin-βA-AS1; IQSEC1—IQ motif and Sec7 domain 1; NPHP4—nephronophthisis 4; PTPRN2—protein tyrosine phosphatase receptor type N polypeptide 2; RASAL1—Ras GTPase-Activating-Like Protein 1; TCF3—GEP100 and transcription factor 3; TGF-β—transforming growth factor-β, TGFBI—TGF, beta-induced.

## Data Availability

Not applicable.
